# The Time-Robustness Analysis of Individual Identification Based on Resting-State EEG

**DOI:** 10.3389/fnhum.2021.672946

**Published:** 2021-09-13

**Authors:** Yang Di, Xingwei An, Wenxiao Zhong, Shuang Liu, Dong Ming

**Affiliations:** ^1^Tianjin International Joint Research Center for Neural Engineering, Academy of Medical Engineering and Translational Medicine, Tianjin University, Tianjin, China; ^2^Lab of Neural Engineering & Rehabilitation, Department of Biomedical Engineering, College of Precision Instruments and Optoelectronics Engineering, Tianjin University, Tianjin, China

**Keywords:** electroencephalography, identification, resting-state, robustness, time-frequency features

## Abstract

An ongoing interest towards identification based on biosignals, such as electroencephalogram (EEG), magnetic resonance imaging (MRI), is growing in the past decades. Previous studies indicated that the inherent information about brain activity may be used to identify individual during resting-state of eyes open (REO) and eyes closed (REC). Electroencephalographic (EEG) records the data from the scalp, and it is believed that the noisy EEG signals can influence the accuracies of one experiment causing unreliable results. Therefore, the stability and time-robustness of inter-individual features can be investigated for the purpose of individual identification. In this work, we conducted three experiments with the time interval of at least 2 weeks, and used different types of measures (Power Spectral Density, Cross Spectrum, Channel Coherence and Phase Lags) to extract the individual features. The Pearson Correlation Coefficient (PCC) is calculated to measure the level of linear correlation for intra-individual, and Support Vector Machine (SVM) is used to obtain the related classification accuracy. Results show that the classification accuracies of four features were 85–100% for intra-experiment dataset, and were 80–100% for fusion experiments dataset. For inter-experiments classification of REO features, the optimized frequency range is 13–40 Hz for three features, Power Spectral Density, Channel Coherence and Cross Spectrum. For inter-experiments classification of REC, the optimized frequency range is 8–40 Hz for three features, Power Spectral Density, Channel Coherence and Cross Spectrum. The classification results of Phase Lags are much lower than the other three features. These results show the time-robustness of EEG, which can further use for individual identification system.

## Introduction

Electroencephalography (EEG), along with the development of neuroscience and computer science, is becoming a new neuroimaging technique that can be used as an alternative method for individual biometric identification (Hema et al., 2008; Chuang et al., 2013). EEG signals reflect individual information about brain anatomy and function, and it can measure the synchronous activity of brain regions (Wolpaw et al., 2000; Rodriguez, 2015). Compared with other biometric identification approaches, such as face, fingerprint, as well as other types of biometric, the EEG-based identification system requires users to be alive and EEG signals are hard to be copied or be hijacked as its sophisticated enough (Wang et al., 2012; Akhtar et al., 2015; Llanos et al., 2019).

Electroencephalography signals were first recorded in 1924 by Hans Berger. The first research on inter-individual variation of EEG signals can track back to 1960s (Davis and Davis, 1936; Berkhout and Walter, 1968), and the relationship between EEG signals and genetic information has been confirmed for the first time (Poulos et al., 1999, 2001, 2002a,b). EEG signals can be quantified by different types of effective measures, such as event-related potentials (ERPs), spectra, functional connectivity as well as other parameters. These time-frequency domain measures can evaluate the inter-individual variability of brain activity. It is not easy to obtain inherent features from raw EEG signals as EEG signals are noisy and small amplitude (Nakanishi et al., 2009; Delpozo-Banos et al., 2015). There are some studies on the EEG-based identification system in recent years. Many analytical methods were used to assess the inter-individual dependence for different types of EEG (Fraschini et al., 2014; Rocca et al., 2014; Alariki et al., 2018). Resting-state is a promising condition used as a biometric for individual identification as it generates synchronous oscillations in specific frequency ranges and compared with other acquisition protocols, it reduces fatigue and artifact since it does not require the active involvement of participants. Lots of studies focus on resting-state of eye open (REO) and closed (REC), and the studies indicated that resting-state EEG carrying interesting information in specific sub-bands have shown significant inter-individual difference especially using related spectral analysis (Abo-Zahhad et al., 2015; Busonera et al., 2018; Chan et al., 2018). Power spectrum of each single electrode can represent the brain oscillation in terms of physiological and cognitive functions (Ramaswamy and Mandic, 2007; Di et al., 2019), and it constitutes inherent information of each region through each channel in different frequency bands (Nakamura et al., 2017). Functional connectivity is another method which captures linear or nonlinear statistical dependencies between distinct channels.

Previous studies pay more attention to the difference of inter-individual variance in one experiment and did not focus on the stability over time for individual identification (Pozo-Banos et al., 2014; Crobe et al., 2016; Zeng et al., 2018). But some features are susceptible to noise that can only be used for intra-experiment data. Therefore, the time-robustness of features used for individual identification is more important when using in the practical identification system (Arnau-Gonzalez et al., 2017; Schetinin et al., 2018).

In this work, we conducted three runs experiments and proposed four feature extraction methods. There are three sessions of REO and REC with time interval of 20 min in each experiment and at least 2 weeks for every two experiment. Support Vector Machine (SVM) was used as the classifier to verify whether the difference between participants and the similarity for different trials of the participant in each run or each fusion run. Then we assessed the stability and time-invariant for individual identification based on inter-run EEG data. Some frequency ranges were chosen to find an optimal frequency range that can obtain a better performance in the frequency range of 1–40 Hz. The results reveal that there is stability and time-robustness of features that we proposed for individual identification based on resting-state EEG data.

## Materials and Methods

### Participants

**FIGURE 1:**
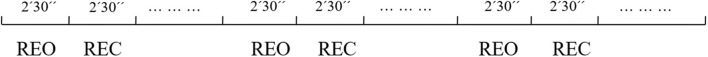
Experimental procedure.

There are 10 participants (6 males) involved in the experiment, with average age of 21(±3). They are volunteers from Tianjin University. Participants have signed the consent form that include notice and individual right before the beginning of first experiment. The study is approved by local ethical committee at Tianjin University. Three sessions are recorded following by 20 min internals in which subjects conduct others protocols. Three run experiments were conducted for each participant and the time interval of runs is at least 2 weeks. The experiment procedure is shown in Figure 1, and the detail of three experiments is shown in Table 1.

**TABLE 1:** Three run experiments date of each subject.

**Number**	**First RUN**	**Second RUN**	**Third RUN**
Sub1	2018/05/25	2018/06/14	2018/07/10
Sub2	2018/05/24	2018/06/14	2018/07/10
Sub3	2018/0527	2018/06/25	2018/07/13
Sub4	2018/05/28	2018/06/26	2018/07/13
Sub5	2018/05/31	2018/06/26	2018/07/16
Sub6	2018/06/02	2018/07/01	2018/07/24
Sub7	2018/06/02	2018/06/28	2018/07/14
Sub8	2018/06/03	2018/06/28	2018/07/15
Sub9	2018/06/04	2018/07/12	2018/08/06
Sub10	2018/06/14	2018/07/11	2018/08/06

### EEG Acquirement

Electroencephalography signals were acquired using the EEG cap with 64 channels placed at the standard position of the international 10–20 system. The channel of “AFz” was set as the ground and the top of head was used as the reference. In this work, there is 20 channels recorded, including Fz, F3, F4, F7, F8, Cz, C3, C4, Pz, P3, P4, PO7, PO8, TP7, TP8, Oz, O1, O2, M1, and M2 (Di et al., 2019).

### Pre-processing

Pre-processing, including down-sampling, re-reference and filtering, is used for EEG data. Firstly, the raw data was down-sampled from 1,000 to 100 Hz, and re-referenced to the mean of ear mastoids ((M1+M2)/2). Then, a bandpass filter of 1–40 Hz was applied. Finally, the data (450s) were epoched into 450 segments (1-s per segment) for each participant in each run.

### Features

#### Power Spectral Density

Power Spectral Density (PSD) is a non-parametric spectrum analysis that describes the distribution of a signal over frequency for stationary random process (Campisi and Rocca, 2015; Wang and Najafizadeh, 2016). The periodogram $\hat{\text{P}}\,\left(\omega \right)$ is defined as:


$$\hat{\text{P}}\,\left(\omega \right)=\frac{\Delta \,\text{t}}{\text{N}}\,{\left|\sum_{n=0}^{N-1}{\text{x}}_{n}\,{\text{e}}^{-\mathrm{j2}\,\pi \,\mathrm{fn}}\right|}^{2},-\frac{1}{2\,\Delta \,\text{t}}<\text{f}\leq \frac{1}{2\,\Delta \,\text{t}}$$

Where x_n_ represents the EEG signal and fn is samples per unit time. **Δ**t is the sampling interval.

The modified periodogram multiplies the series by a window function in order to reduce the leakage in the periodogram. The modified periodogram is defined as:


$$\hat{\text{P}}\,\left(\omega \right)=\frac{\Delta \,\text{t}}{\text{N}}\,{\left|\sum_{n=0}^{N-1}{\text{h}}_{n}\,{\text{x}}_{n}\,{\text{e}}^{-\mathrm{j2}\,\pi \,\mathrm{fn}}\right|}^{2},-\frac{1}{2\,\Delta \,\text{t}}<\text{f}\leq \frac{1}{2\,\Delta \,\text{t}}$$

Where h_n_ is a suitable window function and △t is the sampling interval.

In this work, we use Welch’s method to estimate the PSD of EEG signal. Welch’s average estimation is a method based on modified periodogram. It divides the signal into overlapping segments and averages the estimates that are computed by modified periodogram. This method reduce variance of periodogram by averaging. Hamming Window was used and overlap was set as 0.5. The number of FFT is set as 100 (frequency sampling of signal is 100 Hz). Each segment was characterized by feature vector of PSD, which the size is *N*_*ch*_×*N*_*f*_. *N*_*ch*_ = 18 represent the number of channels we used and *N_f=40* represent the frequency points from 1 to 40 Hz. There are 450 feature vectors of PSD for each participant in each run.

#### Cross Spectrum Analysis

In this part, we estimate the spectral connectivity between channels and compute three features, amplitude spectrum, channel phase lag and channel coherence, to describe the spectrum connectivity between channels (Ghorbanian et al., 2013; Valizadeh et al., 2019). Cross spectrum is a frequency analysis of cross-correlation between two time series. The cross power spectral density is the distribution of power per unit frequency. It is defined as:


$${\text{P}}_{\mathrm{xy}}\,\left(w\right)=\sum_{m=-\infty }^{\infty }{\text{R}}_{\mathrm{xy}}\,\left(m\right)\,{\text{e}}^{-J\,\omega \,m}$$

Where *R*_*xy*_(*m*) is cross-correlation sequence and is defined as:

The complex cross spectrum is obtained through each channel pair. Then we compute the amplitude spectrum and phase lag respectively. The size of amplitude spectrum for each segment is *N*_*p*_×*N*_*f*_, where *N_p=171* means all channel pairs and *N_f=40* means the frequency points from 1 to 40 Hz. There are 450 feature vectors of amplitude spectrum for each participant in each run. The size of phase lag is as same as the size of amplitude spectrum. There are 450 feature vectors of phase lag for each participant in each run.

Coherence estimate is a function which describes how well x corresponds to y in each frequency, with values 0 to 1. *P*_*xy*_ is cross power spectral density and *P*_*xx*_,*P*_*yy*_ are power spectral density.

The coherence is defined as:


$$\text{Cxy}\,\left(f\right)=\frac{{\left|{\text{P}}_{\mathrm{xy}}\,\left(f\right)\right|}^{2}}{{\text{P}}_{\mathrm{xx}}\,\left(f\right)\,{\text{P}}_{\mathrm{yy}}\,\left(f\right)}$$

Where *x* and *y* represent two channels EEG data. The result shows the correlation between two channels at each frequency.

The size of channel coherence is *N*_*p*_×*N*_*f*_, where *N_p=153* represents all channel pairs between channels (exclude self-channel coherence) and *N_f=40* represents the frequency range from 1 to 40 Hz.

#### Pearson Correlation Coefficient

Pearson correlation coefficient (PCC) is a statistic method that can measure the correlation between two variables X and Y. Given a pair of variables X and Y, the PCC is defined as:


$$\rho_{X,Y}=\frac{\text{cov}\,\left(X,Y\right)}{\sigma_{X}\,\sigma_{Y}}=\frac{\text{E}\,\left[\left(\text{X}-\mu_{X}\right)\,\left(\text{Y}-\mu_{Y}\right)\right]}{\sigma_{X}\,\sigma_{Y}}$$

Where *cov* is the covariance,σ*X* is the standard deviation of X and σ_*Y*_ is the standard deviation of Y. μ is the mean and *E* is the expectation.

#### Support Vector Machine

Support Vector Machine (SVM) is a supervised learning method for classification or regression in machine learning (Chang and Lin, 2011; Hong et al., 2013). We are given a dataset of n points X = {*X*_1_,*X*_2_,⋯,*X*_*n*_} and class labels Y = {*y*_1_,*y*_2_,⋯,*y*_*n*_}, where Y ∈ { + 1,−1}, indicating the class of point X. The hyperplane is to divide the group of points *X_i* for which *y*_*i*_ = 1 from the group of points *X_i* for which *y*_*i*_ = −1. It is defined as:


$$\omega^{T}\,{\text{x}}_{k}+\text{b}={\text{y}}_{k}$$

Where ω represent the vector of the hyperplane.

Support Vector Machine is a maximum-margin classifier so we can select two hyperplanes that separate the two classes of data. These two hyperplanes can be described as:


$$\omega^{T}\,x+b=1$$

$$\omega^{T}\,x+b=-1$$

The distance between two hyperplanes is $\frac{2}{||\omega ||}$. In order to maximum the distance between the hyperplanes, we can minimum ω. It can be described as:


$$\max_{\omega ,b}\frac{2}{||\omega ||}$$

$$s.t.{\text{y}}_{\text{i}}\,\left(\omega^{\text{T}}\,{\text{x}}_{\text{i}}+b\right)>0$$

the paradigm is based on PsychtoolBox in Matlab and the preprocessing of EEG data is based on EEGLAB in Matlab (Brunner et al., 2013). All programming codes of feature extraction and classification were written in Matlab.

## Results and Discussion

Biometrics is a heated topic and EEG-based biometric system which draw more attention in a few years. Although there are some researches about the EEG-based biometrics system, most of them just focus on the difference between participants in a single experiment, and ignore the stability and time-robustness of inter-experiments data independently (Koike-Akino et al., 2016; Wu et al., 2018; Özdenizci et al., 2019), which is much more important.

In this section, the relevant results are shown for all participants based on resting-state (REO and REC) EEG signals. Both four features extraction approaches which are described in Section-II are used in this section to investigate the stability of intra-run and inter-runs features. Figures of extracted features are visible in Section III-1, and related classification results for inter-run and intra-runs features are showed in Section III-2 and III-3. Moreover, our mainly goal is to assess the stability and reliability of EEG features. We estimate spectral information of each single channel and functional connectivity with channel pairs by different feature extraction methods according to the previous works that spectral density of single channel and coherence measures of channel pairs can be useful features for identification with high accuracy (Rocca et al., 2014; Di et al., 2019; Valizadeh et al., 2019). In this work, we used the approaches which were given in Section II to obtain the features, and randomly selected 4 participants from all 10 participants to show the difference of features, visually. The method of PCC is used to measure the linear correlation for each feature, and the classifier of SVM is used to obtain the classification accuracy.

### Features

In this part, values of four features of each participant are presented to show the difference. Power Spectral Density, Cross Spectrum, Phase Lags, and Channel Coherence, ξ_*PSD*_,ξ_*spectrum*_,ξ_*phase*_ and ξ_*COH*_, are obtained refer to previous methods in Section II. In this work, each feature has 450 trials for REO and REC, and in order to reduce the noise, 90 trials for each condition of each participant were obtained by averaging every five trials. The intra-run coefficients are also calculated in this part for correlation analysis. Here we use Fisher’s Z transformation to the Channel Coherence and logarithmic transformation to the PSD and Cross Spectrum values (Valizadeh et al., 2019).

**FIGURE 2:**
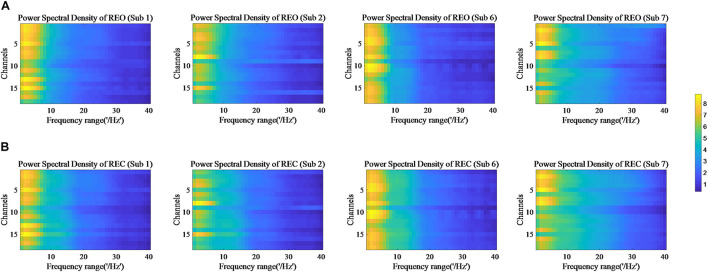
Power Spectral Density of REO and REC for four subjects. **(A)** Shows the condition of REO. **(B)** Shows the condition of REC.

**FIGURE 3:**
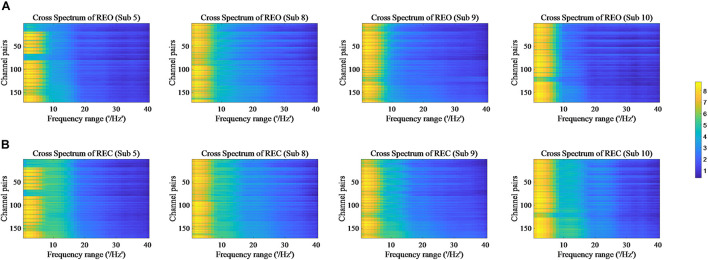
Cross Spectrum of REO and REC for four subjects. **(A)** Represents the condition of REO. **(B)** Represents the condition of REC.

**FIGURE 4:**
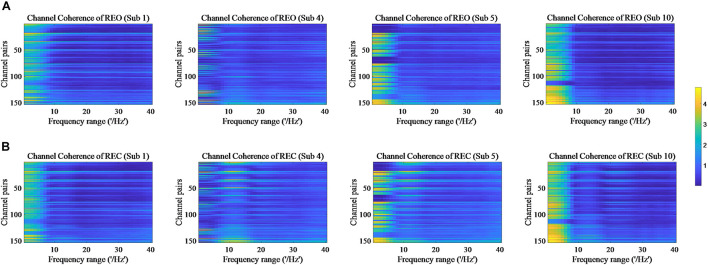
Channel Coherence of REO and REC for four subjects. **(A)** The features of REO. **(B)** The features of REC.

**FIGURE 5:**
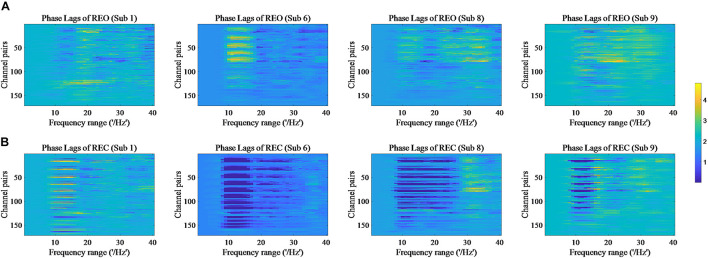
Phase Lags of REO and REC for four subjects. **(A)** Represents the condition of REO. **(B)** Represents the condition of REC.

The values of PSD, Cross Spectrum, Channel Coherence, and Phase Lags are visible in Figures 2–5, respectively. Four participants were randomly chosen for each feature. The *X*-axis represents the frequency range from 1 to 40 Hz, and *Y*-axis represents each single channel or channel pairs. The upper and bottom in Figures 2–5 show the condition of REO and REC, respectively. The change in color from yellow to blue corresponds to change of value from large to small. Power Spectral Density can reflect the brain activity for the position of EEG channels over scalp. All 18 channels are calculated for PSD. Cross Spectrum, Phase Lags, and Channel coherence can reflect functional connections of channel pairs. In this work, we get 171 channel pairs overall, with frequency ranges from 1 to 40 Hz, for Cross spectrum and Phase Lags of each participant, and 153 channel pairs (exclude 18 self-channel pairs), with 1–40 Hz, for Channel Coherence of each participant.

The values of PSD for participants on REO and REC are visible in Figure 2. As we can see, there is a numerical difference between participants for REO and REC, respectively. The values of 1–10 Hz are higher than other frequency ranges for each channel of REO, and for REC, the values of 1–15 Hz are higher than other frequency ranges. Figures 3, 4 show similar conclusion. The feature values of 1–10 Hz are much higher than other frequency ranges for REO and REC, and for each figure of the same participant, from figures, we can see that a little less difference between REO and REC, except the frequency range of 10–15 Hz, in which the values of REC are much higher than that of REO. As for the feature of Phase Lags, there is distinct between participants, and the values of frequency range around 10 Hz are positive for REO, in which the values are negative in the same frequency range for REC.

**FIGURE 6:**
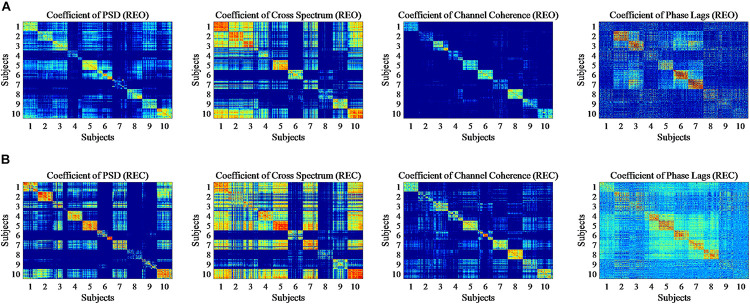
Pearson Correlation coefficient (PCC) for 90 trials of each subject in single run based on REO and REC. **(A)** Represents the condition of REO. **(B)** Represents the condition of REC.

The above shows the difference of intra-run data visually and statistically. Moreover, Pearson correlation coefficients (PCC) are calculated to show whether features of intra-subject have the similarity and features of inter-subjects have the difference statistically, respectively. In this part, as before, every five trials of each feature were averaged and finally got 90 averaged trials. The PCC results of PSD, Cross Spectrum, Channel Coherence, and Phase Lags are visible in Figure 6. The *X*-axis and *Y*-axis represent trials for all participants of the same experiment, and number of 1 to 10 represent the subject number. The coefficient values are ranged from −1 to +1, in which close to ‘0’ represents lower correlation and close to “(±)1” represents higher correlation (positive or negative) of intra-run. The upper in Figure 6 shows the condition of REO. The bottom of Figure 6 shows coefficients for the condition of REC. To show the contrast significantly, the minimum values of figures were changed.

From the figures we can see that the diagonal of each figure, which means the intra-run correlation coefficients for each subject, shows a more significant correlation than the correlation of different subjects, although four features show the correlation of intra-run data in a different level. It seems that the correlation of two features, PSD and Channel Coherence, is more significant than the other two features, and the correlation of Phase Lags is less more significant in four features for intra-run data.

### Classification Results

In this part, the classification results are shown using SVM as the classifier. The 10-fold cross-validation is used to obtain the average accuracies. Three runs are defined as RUN1, RUN2, and RUN3, respectively, and we also define four fusion runs which consist of three experiment data as F-RUN, in which F-RUN1 consist of data of RUN1 and RUN2, F-RUN2 consist of data of RUN1 and RUN3, F-RUN3 consist of data of RUN2 and RUN3, F-RUN4 consist of data of RUN1, RUN2, and RUN3. We divide the F-RUN into two sets, train set and test set, which both include part of two or three runs data.

#### Intra-Run

Table 2 shows the classification results of four features comprised PSD, Cross Spectrum, Channel Coherence, and Phase Lags, for two protocols of REO and REC, to investigate the stability for intra-run and fusion-runs.

**TABLE 2:** The classification results.

**Protocols**	**Features**	**RUN1**	**RUN2**	**RUN3**	**F-RUN1**	**F-RUN2**	**F-RUN3**	**F-RUN4**
REO	PSD	96.3%	97.4%	98.6%	97.6%	98.2%	97.5%	96.9%
	Cross Spectrum	98.9%	100%	100%	100%	100%	100%	100%
	Channel coherence	100%	100%	100%	98.3%	100%	100%	100%
	Phase Lags	96.7%	96.9%	96.6%	97.3%	94.8%	94.8%	94.9%
REC	PSD	98.3%	98.6%	97.9%	99.8%	100%	99.2%	99.8%
	Cross Spectrum	100%	100%	99.6%	99.8%	99.8%	100%	99.6%
	Channel Coherence	98.6%	100%	99.2%	98.9%	98.6%	99.3%	98.1%
	Phase Lags	90.1%	94.2%	87.6%	88.6%	83.5%	88.7%	86.3%

*The classification results show in this table, for four features of each single run and fusion run on REO and REC.*

The classification results of intra-run and fusion-runs data are obtained using SVM. The results revealed in Table 2. The lowest accuracy can reach 80% and the highest accuracy can reach 100%. The accuracies of three features, PSD, Cross Spectrum and Channel Coherence, are approximately equal for intra-run or fusion-runs data on REO and REC. The classification results of Phase Lags based on REC for fusion-runs data, which only reach 80%, are lowest in the table, compared with other results. From the results of the table, given the interfere of noise, it seems that the features we used in this work are distinct for intra-run and fusion-runs data between different subjects.

#### Inter-Runs

The primary task of this work is to assess the stability and time-robustness of each feature we used for inter-runs EEG data. Further, we test the features of inter-runs respectively.

In this part, we mainly show the results of inter-runs classification. Here we define three conditions and investigate the time-robustness and stability of inter-runs features, independently. The conditions are: (1) Using RUN1 and RUN2 as train set and validation set, and RUN3 as test set; (2) Using RUN1 and RUN3 as train set and validation set, and RUN2 as test set; and (3) Using RUN2 and RUN3 as train set and validation set, and RUN1 as test set. We named these as COND1, COND2, and COND3, respectively, and use the abbreviations in the content behind.

The classifier of SVM is used for all three conditions to show the stability of inter-runs features. The classification results of different features for inter-runs data, which are based on REO and REC, are visible in Tables 3–6. In this part, 13 frequency ranges were chosen as shown in tables. Four familiar frequency ranges refer to brain activity are used, including θ (4–7 Hz), α(8–13 Hz), β(13–20 Hz, 20–30 Hz), and a part of γ (30–40 Hz). The classification results of some combined ranges, including 4–20 Hz, 4–30 Hz, 8–20 Hz, 8–30 Hz, 8–40 Hz, 13–30 Hz, and 13–40 Hz, are calculated, and the classification result of original range (1–40 Hz) is also calculated as a benchmark compared with the results of others.

**TABLE 3:** Classification results of PSD for different ranges on REO and REC.

**Frequency**	**REO (%)**			**REC (%)**		
**Range (Hz)**	**COND1**	**COND2**	**COND3**	**COND1**	**COND2**	**COND3**
4–7	1	18	20	1	10	16
8–13	34	31	34	28	43	41
13–20	71	73	84	67	65	71
20–30	69	78	72	66	69	75
30–40	59	77	68	62	71	68
4–20	25	34	44	12	23	28
4–30	49	54	61	36	38	45
8–20	60	59	73	63	64	69
8–30	71	75	82	74	76	83
8–40	77	81	80	74	83	83
13–30	76	81	84	71	71	81
13–40	80	84	83	75	82	82
1–40	51	55	62	25	33	44

**TABLE 4:** Classification results of cross spectrum for different frequency ranges on REO and REC.

**Frequency**	**REO (%)**			**REC (%)**		
**Range (Hz)**	**COND1**	**COND2**	**COND3**	**COND1**	**COND2**	**COND3**
4–7	2	10	10	2	10	7
8–13	28	27	39	45	49	64
13–20	75	75	88	70	74	79
20–30	74	79	76	77	77	79
30–40	70	80	75	70	67	68
4–20	24	29	44	21	22	28
4–30	46	48	58	30	34	48
8–20	60	53	59	66	68	28
8–30	73	74	81	78	80	86
8–40	74	74	81	81	82	82
13–30	78	79	85	77	81	80
13–40	80	81	86	78	84	86
1–40	47	54	57	20	28	43

**TABLE 5:** Classification results of channel coherence for different frequency ranges on REO and REC.

**Frequency**	**REO (%)**			**REC (%)**		
**Range (Hz)**	**COND1**	**COND2**	**COND3**	**COND1**	**COND2**	**COND3**
4–7	14	2	12	7	8	11
8–13	29	29	37	56	50	67
13–20	58	61	73	63	64	79
20–30	63	77	70	54	72	74
30–40	63	79	73	60	73	62
4–20	20	17	26	21	18	34
4–30	29	32	43	27	32	44
8–20	49	48	60	66	61	74
8–30	69	68	70	76	75	81
8–40	73	76	72	74	83	83
13–30	69	76	77	69	76	77
13–40	70	79	76	68	82	81
1–40	34	36	44	25	28	35

**TABLE 6:** Classification results of phase lags for different frequency ranges on REO and REC.

**Frequency**	**REO (%)**			**REC (%)**		
**Range (Hz)**	**COND1**	**COND2**	**COND3**	**COND1**	**COND2**	**COND3**
4–7	12	16	11	7	14	14
8–13	26	33	34	56	40	47
13–20	31	31	35	46	51	54
20–30	25	27	28	37	40	39
30–40	24	37	27	30	32	31
4–20	33	35	40	45	51	53
4–30	35	40	40	49	54	54
8–20	33	35	39	46	52	56
8–30	35	40	42	50	56	57
8–40	38	46	46	49	53	52
13–30	35	36	40	47	50	53
13–40	37	43	42	50	58	55
1–40	28	41	24	35	60	39

Table 3 shows the classification results of inter-runs PSD for REO and REC. As we can see that the results of the frequency range of 4–7 Hz are lowest (1, 18, and 20%) for three conditions on REO and REC, and the highest average result is at the frequency range of 13–40 Hz on REO, which can reach up to 84%. The results at 4–7 Hz, 8–13 Hz, and 4–20 Hz are lower than the results at 1–40 Hz, and the results of 4–30 Hz are equal to the results at 1–40 Hz, approximately. The results of the frequency range at 13–20 Hz, 20–30 Hz, and 30–40 Hz are higher than the results of 1–40 Hz, which means that the frequency ranges of these three ranges consist of inherent information about the difference between participants. Next, compared with the results of 4–20 Hz and 4–30 Hz, the results of frequency ranges at 8–20 Hz and 8–30 Hz are significantly increased. Considering the poor results of the frequency range at 4–7 Hz, it is believed that the frequency range at 4–7 Hz of PSD does not have the stability for identification. Compared with the results of frequency ranges at 8–30 Hz and 8–40 Hz, the results we obtained at 13–30 Hz and 13–40 Hz have increased. Therefore, we think that the frequency range of PSD that contains more stability information for inter-run data is 13–40 Hz. The optimized frequency range is at 13–40 Hz for REO, in which the average accuracy can reach 82.33%.

As for REC, the lowest accuracies are at 4–7 Hz, which are 1, 10, and 16%, for three conditions, respectively, and the highest average accuracies are at 8–40 Hz, which can reach 80%. Compared with the results of the frequency range at 1–40 Hz, the results of other frequency ranges, exclude frequency range at 4–7 Hz and 4–20 Hz, are little or much higher. The results of two frequency ranges, 8–30 Hz, and 8–40 Hz, are higher than then results of frequency ranges at 13–30 Hz and 13–40 Hz, which are higher than the results of 4–20 Hz and 4–30 Hz. Like the results of REO, the frequency range of 4–7 Hz contain less information about the stability for inter-runs feature of PSD, but other than the results of REO, the frequency range of 8–13 Hz seems to be related to inherent information for identification. Therefore, the classification results of REC show that it seems the frequency range at 8–40 Hz contains much information that can be used as an optimized frequency range of PSD for identification.

Table 4 reveals the classification results of Cross Spectrum for inter-runs on REO and REC. From the table we can see that the accuracies of the frequency range at 1–40 Hz are much lower, which are around 50% for REO and around 30% for REC. For the results of REO, the lowest accuracies are at 4–7 Hz, which are 2, 10, and 10% for three conditions, respectively, and the highest average result is at frequency range of 13–40 Hz, which can reach 82.33%. The results of frequency ranges at 13–30 Hz and 13–40 Hz are higher than the results at 8–30 Hz and 8–40 Hz, which are higher than the results of 4–30 Hz. Like the results of PSD on REO, the frequency ranges of 4–7 Hz and 8–13 Hz of inter-runs are not suitable for individual identification. It seems that the frequency range of 13–40 Hz is an optimized range that can be used for inter-runs classification.

For classification results of Cross Spectrum on REC, the lowest results are at 4–7 Hz, which are 2, 10, and 7%, respectively. The highest results are at 8–40 Hz, which is as same as the frequency range of PSD on REC. The accuracies of Cross Spectrum on REC at 8–30 Hz and 8–40 Hz are higher than the results of frequency ranges at 13–30 Hz and 13–40 Hz, which are higher than the results of frequency range of 4–20 Hz and 4–30 Hz. Therefore, like the conclusion we obtained from PSD of REC, the frequency range at 8–40 Hz is an optimized range for inter-runs identification, which is much higher than the results at 1–40 Hz that the accuracies are only 20, 28, and 43% for three condition, respectively.

The classification results of Channel Coherence are visible in Table 5. As we can see that the highest accuracy can achieve 79% for REO, and 83% for REC. The lowest accuracy is less than 10% for REO and REC. The classification accuracy is lower when frequency range include the range of 4–7 Hz, such as 1–40 Hz, 4–20 Hz, 4–30 Hz, and 4–7 Hz. The result of 4–7 Hz is lowest than results of other frequency ranges. The results of 4–20 Hz and 4–30 Hz are significantly lower than results of 8–20 Hz and 8–30 Hz. These results show that frequency range of 4–7 Hz contain more irrelevant information than other frequency ranges for REO and REC.

For classification results of REO, the results of three frequency ranges, which are, 13–20 Hz, 20–30 Hz, and 30–40 Hz, are higher than frequency range of 1–40 Hz for three conditions, and it seems that each of these frequency ranges may contains part of information about individual stability and time-invariant. Results of combined frequency ranges (13–30 Hz and 13–40 Hz) show higher classification performance than other frequency ranges, which can reach 80% for three conditions, Therefore, there is no doubt that 13–40 Hz is a more appropriated frequency range of REO for inter-runs classification of Channel Coherence which can be used in individual identification. For classification results of REC, the highest average accuracy is at frequency range of 8–40 Hz, which can reach 80%, and the lowest accuracy is at 4–7 Hz. The frequency range of 8–13 Hz for REC seems contain some more related information about stability and time-invariant than that for REO. The appropriate optimized frequency range of REC is 13–40 Hz.

The classification results of Phase Lags show in Table 6. The results of all chosen frequency ranges show poor performance for inter-runs classification, and the highest accuracy only reach 60%, which is much lower than the classification results of other three features. Unlike the other three features, optimization of frequency range cannot get a satisfied performance for inter-runs classification. The classification results obtained for inter-runs data also much lower than the results we obtained for intra-run and fusion-runs classification, which can reach 80% or higher. Therefore, it seems that Phase Lags is not the useful feature of inter-run data for individual identification.

There are some limitations in this study. First, the number of sample size is relatively small. Considering it is a pilot study, the further study needed to verify the reliability of results. Second, the sex differences may influence the results and it will be investigated with extending the number of sample size in the further study.

## Conclusion

In this paper, we mainly analyze the stability and time-robustness of resting-state EEG features for individual identification. The number of participants is 10 and three runs are conducted for each participant. The time interval between each experiment is at least 2 weeks.

The results show that:

(1) The similarity of intra-individual and the difference of inter-individual for intra-run features based on REO and REC. Perfect classification results for intra-run and fusion-runs features on REO and REC.

(2) For inter-runs features classification of REO, the optimized frequency range is at 13–40 Hz for three features, which are PSD, Cross Spectrum and Channel Coherence. For inter-runs features classification of REC, the optimized frequency range is at 8–40 Hz for three features, which are PSD, Cross Spectrum and Channel Coherence. The classification results of Phase Lags are poor for REO and REO, and it seems not to be used for individual identification.

(3) The results suggested that features of PSD, Channel Coherence and Cross Spectrum are stability and time-invariant that can be used for individual identification and will help to develop a more stable identification system based on EEG data.

## Data Availability Statement

The raw data supporting the conclusions of this article will be made available by the authors, without undue reservation.

## Ethics Statement

The studies involving human participants were reviewed and approved by Tianjin University. The patients/participants provided their written informed consent to participate in this study. Written informed consent was obtained from the individual(s) for the publication of any potentially identifiable images or data included in this article.

## Author Contributions

All authors listed have made a substantial, direct and intellectual contribution to the work, and approved it for publication.

## Conflict of Interest

The authors declare that the research was conducted in the absence of any commercial or financial relationships that could be construed as a potential conflict of interest.

## Publisher’s Note

All claims expressed in this article are solely those of the authors and do not necessarily represent those of their affiliated organizations, or those of the publisher, the editors and the reviewers. Any product that may be evaluated in this article, or claim that may be made by its manufacturer, is not guaranteed or endorsed by the publisher.
